# Heartburn Takes a Whole New Meaning: Multidisciplinary Management of a Gastropericardial Fistula

**DOI:** 10.1155/cris/5597348

**Published:** 2026-05-13

**Authors:** Jake Sypniewski, Jesse K. Kelley, G. Dane Fritz, Geoffrey Lam, Eugene Zolotarevsky

**Affiliations:** ^1^ College of Human Medicine, Michigan State University, Grand Rapids, Michigan, USA, msu.edu; ^2^ Department of General Surgery, Corewell Health West Michigan, Grand Rapids, Michigan, USA; ^3^ Division of Cardiothoracic Surgery, Corewell Health West Michigan, Grand Rapids, Michigan, USA; ^4^ Department of Gastroenterology, Corewell Health West Michigan, Grand Rapids, Michigan, USA

**Keywords:** endoscopic stenting, gastropericardial fistula, pericardial window, video-assisted thoracoscopic surgery (VATS)

## Abstract

Gastropericardial fistulas (GCs) are exceedingly rare in the reported literature. Etiologies include prior abdominal surgery, malignancy, and perforated gastric ulcers. Presenting symptoms range from chest pain to cardiac tamponade. Early imaging can help delineate the origin of the fistula. Management involves a multidisciplinary team, involving endoscopic stenting for temporization followed by definitive surgery for fistula closure. Robotic, video‐assisted thoracoscopic surgery (VATS) with pericardial window has only been reported once in modern literature as definitive management for GCs. Thus, we present a case of a gastro‐pericardial fistula that was temporized with endoscopic stenting and surgically managed with right robotic VATS with a pericardial window and definitive fistula closure. The patient had presented in cardiac tamponade with initial imaging demonstrating a large, loculated hydropneumopericardium and GC. The patient’s postoperative course was unremarkable, and she was discharged on postoperative day 7. Follow‐up within our health system is currently 4.5 years, and she continues to do well.

## 1. Introduction

A gastropericardial fistula (GC) is a rare but life‐threatening, aberrant connection between the stomach and pericardium. Given its rarity, there is a paucity of data in the literature. A recent literature search identified only 32 reports of GC since 2000 [1]. Etiologies described include prior abdominal surgery, perforated gastric ulcers, malignancy, foreign body, and caustic ingestion. In particular, a history of foregut surgery with subsequent ulcer formation was reported in one third of patients with GC [1]. Surgical correction of hiatal and diaphragmatic hernias has also been reported as a nidus for fistula formation. The clinical presentation is nonspecific and can include chest pain, epigastric pain, dyspnea, fever, dysphagia, emesis, or hematemesis. A high index of suspicion is needed to ensure early detection, which results in improved survival in this patient population [1]. Herein, we describe a case of a female in her mid‐50s presenting with cardiac tamponade secondary to an underlying GC who was successfully treated via a multidisciplinary approach.

## 2. Clinical Presentation

**Figure 1:**
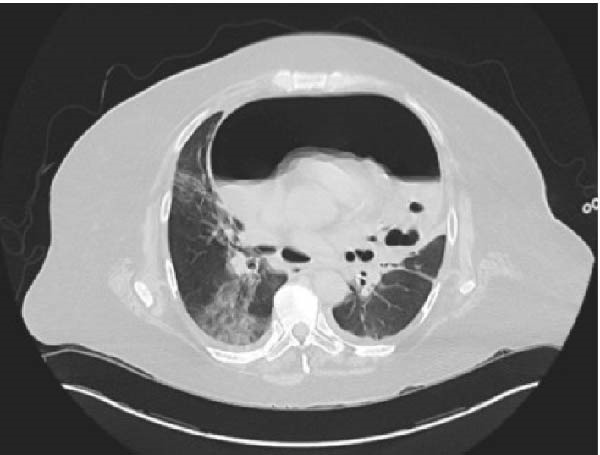
Transverse slice from CT imaging demonstrating large loculated hydropneumopericardium.

**Figure 2:**
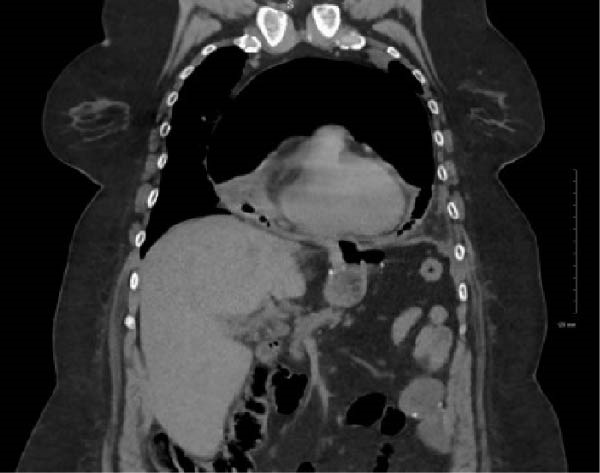
Coronal slice from CT imaging demonstrating large loculated hydropneumopericardium.

A female in her mid‐50s with a known gastro‐gastric fistula presented to an offsite emergency department with chest pain. She had associated neck and back pain, along with exertional dyspnea for 4 days prior to presentation. Her medical history was significant for active tobacco use and a 35 pack‐year history, hypertension, and recurrent marginal ulcers on twice daily proton pump inhibitors and Carafate. Relevant surgical history included an open Roux‐en‐Y gastric bypass years prior and recurrent upper gastrointestinal bleeds requiring angioembolization of a left phrenic artery pseudoaneurysm. On examination, the patient was hypotensive and tachycardic with a friction rub heard on cardiac auscultation. Electrocardiogram (EKG) demonstrated ST elevations in the inferior leads and generally low voltage, but she had marginally elevated and adynamic troponins on recheck, less concerning for type 1 myocardial infarction. Computed tomography (CT) of the thorax, abdomen, and pelvis showed an extensive loculated hydropneumopericardium (Figures 1 and 2). The patient’s EKG abnormalities were attributed to stress‐induced myocardial ischemia and pericardial effusion given the hemodynamic instability and imaging findings of a large pericardial effusion. An emergent pericardial drain was placed, and she was transferred to our institution for further evaluation and management. Upon arrival, empiric Zosyn and micafungin were initiated, and the gastroenterology and thoracic surgery teams were consulted, and a staged approach for management was planned.

**Figure 3:**
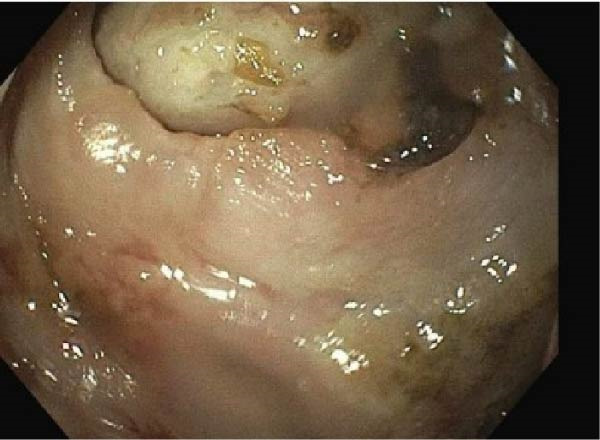
Endoscopic image of the gastropericardial fistula originating from the gastrojejunostomy limb.

A gastrografin esophagram was obtained and was negative for esophageal perforation. An upper gastrointestinal series (UGI) subsequently demonstrated an irregular collection of contrast extending superior to the gastric pouch. Gastroenterology performed an upper endoscopy on hospital day 2, which revealed a 1.3 cm gastro‐jejunal ulcer with fistula tract to the pericardium (Figure 3). The gastroenterology team placed a 15 cm x 1.8 cm self‐expanding metal stent to exclude the fistula tract from the alimentary tract. On hospital day 2, the patient inadvertently removed the pericardial drain that was placed. At that time, the patient was hemodynamically stable and there were no emergent concerns for acute surgical intervention on that day. Thus, on the following hospital day 3, the patient was taken for a right robotic video‐assisted thoracoscopic surgery (VATS) with plans for pericardial washout and definitive GC closure.

**Figure 4:**
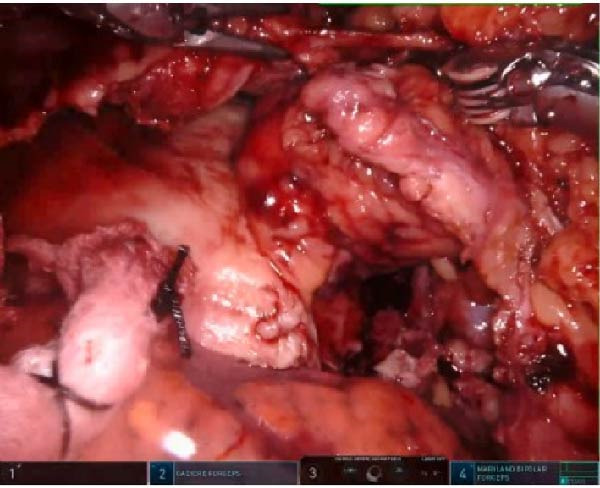
Intraoperative image of robotic‐assisted thoracoscopic washout and repair of fistula.

Intraoperatively, there was evidence of acute pericarditis, and on entry into the pericardium, 350 mL of straw‐colored fluid was aspirated and sent for culture analysis, which returned positive for *Streptococcus pneumonia*e. Loculations within the pericardial space were disrupted and washed out (Figure 4). A pericardial fat pad was mobilized, buttressed over the fistula tract, and secured in place, and a pericardial drain was placed adjacent to the fistula. The patient tolerated the procedure without issue and did not require ICU‐level care. Her postoperative course was relatively unremarkable with minimal serosanguinous drainage from the pericardial drain. The pericardial drain was removed at bedside on postoperative day 3, and no subsequent leak was detected. Follow‐up EKG failed to redemonstrate ST changes as previously described. She was transitioned to Unasyn and fluconazole postoperatively and transitioned to oral Augmentin and fluconazole to complete a planned 4‐week course outpatient. The patient was discharged on postoperative day 7 in stable condition and advised to continue sucralfate, pantoprazole, and misoprostol. She was seen by gastroenterology outpatient for a repeat upper endoscopy 6 weeks postprocedure, which demonstrated complete closure of the fistula tract, and the stent was removed. The patient has been followed for 4.5 years within our health system and continues to do well.

## 3. Discussion

GCs are exceedingly rare in the modern era, with a prior case report highlighting 32 cases reported in the literature [2]. The first report of a GC was published in 1863 and was secondary to a subphrenic abscess related to gastric ulcers [3]. Advanced imaging now plays an integral role in the diagnosis of this rare pathology. Chest radiographs are often ordered with symptoms of chest pain and dyspnea; however, they are nondiagnostic in the setting of a GC but can demonstrate pneumopericardium. CT imaging with intravenous contrast is often obtained to further assess the etiology of the patient’s nonspecific symptoms. Oftentimes, this is not obtained with oral contrast, which limits the ability to identify a fistula tract; however, the clinician should maintain a high index of suspicion in the setting of pneumopericardium [4]. Instead, a UGI utilizing gastrografin can better demonstrate the anatomy and location of the fistula tract if present [5]. In addition, an esophagogastroduodenoscopy (EGD) has also been employed to localize the GC, which allows stent placement to temporarily exclude the fistula until definitive surgery can be performed [4, 6].

After diagnosis, prompt treatment with source control is required to prevent further clinical deterioration. Empiric antibiotics following one’s institutional nomogram should be initiated given the connection of the gastrointestinal tract to the pericardium. Cultures should be obtained as soon as able to allow for more directed antibiotic therapy. Surgical intervention can be utilized for closure of the fistula. As previously mentioned, therapeutic endoscopy can also be utilized, both for temporizing measures as well as definitive treatment. When surgery is pursued, both minimally invasive and open transthoracic and abdominal approaches have been described [4–7]. In our case, there has been only one other case reported highlighting the use of VATS for intraoperative, definitive fistula closure [8]. In addition, despite self‐removal of the pericardial drain, the patient was hemodynamically stable and did not warrant any acute surgical intervention with an open approach. It was deemed appropriate by the surgical team to intervene on the following day with the less invasive, robotic VATS approach. Compared to thoracotomy, VATS is beneficial in allowing for real‐time video of the intervention along with smaller incision sizes.

Regardless of intervention, the prognosis of those suffering from GC is typically poor due to delayed diagnosis [2, 9]. Fortunately, the morbidity and mortality of patients with these fistulas have drastically improved in recent decades secondary to enhanced imaging modalities resulting in earlier detection. Before the year 2000, the average survival rate for patients with a GC was estimated at 31%; since then, it has increased significantly to 89%. The predominant etiology has also transitioned over this time period with peptic ulcer disease being responsible for 75% of these fistulas prior to the year 2000, to foregut surgery now being responsible for the majority (65%) [1]. Interestingly, only one case report has documented *Helicobacter pylori* perforated gastric ulcer as a nidus for GC evolution [10]. In summary, this case report demonstrates successful, multi‐disciplinary endoscopic and surgical management of an exceedingly rare clinical pathology.

## Funding

No funding was received for this research.

## Conflicts of Interest

The authors declare no conflicts of interest.

## Data Availability

Data sharing is not applicable to this article, as no datasets were generated or analyzed during the current study.
